# Investigation of protective effects of rutin\cyclodextrin inclusion complex against testicular damage caused by diisononyl phthalate in rats

**DOI:** 10.22038/ijbms.2025.87549.18910

**Published:** 2025

**Authors:** Ramazan Bozali a, Serkan Ali Akarsu, Cihan Gür, Sefa Küçükler, Nurhan Akaras, Mustafa İleritürk, Serhat Sunar, Fatih Mehmet Kandemir

**Affiliations:** 1 Department of Reproduction and Artificial Insemination, Faculty of Veterinary Medicine, Atatürk University, Erzurum, Turkey; 2 Department of Medical Laboratory Techniques, Vocational School of Health Services, Atatürk University, Erzurum, Turkey; 3 Department of Biochemistry, Faculty of Veterinary Medicine, Atatürk University, Erzurum, Turkey; 4 Department of Histology and Embryology, Faculty of Medicine, Aksaray University, Aksaray, Turkey; 5 Department of Laboratory and Veterinary Health, Horasan Vocational School, Atatürk University, Erzurum, Turkey; 6 Department of Reproduction and Artificial Insemination, Faculty of Veterinary Medicine, Kafkas University, Kars, Turkey; 7 Department of Medical Biochemistry, Faculty of Medicine, Aksaray University, Aksaray, Turkey

**Keywords:** DINP, Oxidative stress, Rutin\cyclodextrin, Sperm quality, Testicular damage

## Abstract

**Objective(s):**

The aim of this study was to investigate the protective effects of Rutin\cyclodextrin (RUT\CD) complex in rats exposed to diisononyl phthalate (DINP).

**Materials and Methods:**

In the study, 35 male Sprague Dawley rats were used. The rats were randomly divided into five groups: Control, DINP, RUT\CD, DINP+RUT\CD100, and DINP+RUT\CD200. The control group received Tween 80 by oral gavage, while the DINP groups received DINP at a dose of 200 mg/kg/bw. RUT+CD groups received the RUT\CD complex by oral gavage. After 14 days of administration, rats were sacrificed, and testicular tissues were used for histopathological and biochemical analyses, and epididymal tissues were used for semen analysis.

**Results:**

DINP administration caused an increase in MDA level and a decrease in SOD, CAT, GPx1 enzyme activities, and GSH level in rats. RUT\CD administration decreased oxidative stress and increased antioxidant activity. In addition, DINP administration caused a decrease in Nrf-2 and HO-1 levels. DINP caused a significant increase in eIF2α, ATF4, NF-κB, TNF-α, IL-1β, IL-6, Inos, and Cox2 levels in the testicular tissue of rats. RUT\CD administration decreased these levels in a dose-dependent manner. Apoptosis markers p53, Apaf-1, Bax, Bcl-2, and Caspase-3 mRNA transcript levels and Bax and Bcl-2 protein levels were significantly increased in the DINP-administered group. In the DINP+ RUT/CD group, these levels decreased in a dose-dependent manner. Moreover, DINP administration caused an increase in sperm DNA damage.

**Conclusion:**

DINP administration induced testicular toxicity by increasing oxidative stress, apoptosis, and inflammation, and changes in testicular histology. Moreover, RUT\CD administration attenuated DINP-induced toxic effects.

## Introduction

Phthalates, which are endocrine disrupting chemicals (EDCs), can cause dysfunction in hormonal systems in the body (1). Phthalates are chemical compounds known as plasticizers and are used to add flexibility, strength, and transparency to plastic materials (2). Phthalates are classified according to their molecular weight. Low-molecular-weight phthalates include species such as Dimethyl Phthalate (DMP), Diethyl Phthalate (DEP), Dibutyl Phthalate (DBP), and Butyl Benzyl Phthalate (BBP). On the other hand, high-molecular-weight phthalates, such as Di-(2-ethylhexyl) Phthalate (DEHP) and Di-isononyl Phthalate (DINP), are used in the production of more durable and long-lasting plastic products (3). DINP, as a high molecular weight plasticizer, is widely used to improve the flexibility, durability, and longevity of plastic products, especially PVC-based plastic products (4). DINP is oil-soluble and has a low tendency to evaporate, which improves the performance of plastic materials due to its stable structure; however, this raises concerns about health risks associated with exposure (5). Once in the bloodstream, DINP can accumulate in adipose tissues and produce long-term biological effects (6). It has been reported that DINP has disruptive effects on the endocrine system and especially adversely affects male reproductive health (7).

Rutin (RUT) (quercetin-3-O-rutinoside or 3′,4′,5,7-tetrahy-droxy-flavone-3-rutinoside) is also a flavonoid well known for its anti-inflammatory, antiplatelet, vasoactive, and antihypertensive properties. Antiallergic, antispasmodic, hypolipidemic, cytoprotective, antitumor, antiprotozoal, antibacterial, and antiviral activities (8). RUT plays a crucial role in mitigating oxidative stress in testicular tissue due to its potent antioxidant properties. It limits cellular damage caused by reactive oxygen species (ROS) by directly neutralizing free radicals and protects cell membrane integrity by preventing lipid peroxidation (9). RUT has very low solubility in water due to its hydrophobic nature, which severely limits its bioavailability. It also tends to be rapidly metabolized in the gastrointestinal tract and excreted from the body before reaching the target tissue. These pharmacokinetic limitations reduce the therapeutic efficacy of direct administration of RUT and necessitate the use of appropriate carrier systems to increase its bioavailability (10). In this context, carrier molecules such as cyclodextrin (CD) have the potential to optimize the efficacy of RUT by increasing its solubility, stability, and absorption (11). The RUT\CD complex formed with cyclodextrin optimizes the pharmacokinetic and pharmacodynamic properties of RUT. CD incorporates RUT molecules into its internal cavity, increasing its solubility and dissolution rate. Increased solubility increases bioavailability (12, 13).

The aim of this study was to determine the effects of RUT\CD complex against DINP-induced testicular toxicity by biochemical, histopathological, and spermatological analyses.

## Materials and Methods

### Chemicals

DINP (CAS no: 28553-12-0) was obtained from Sigma-Aldrich chemical (St. Louis, MO, USA). RUT (CAS no: 250249-75-3) was purchased from Cayman Chemical Company (Cayman Chemical Company, USA). B-Cyclodextrin (CAS No.: 7585-39-9) was purchased from BLD Pharm (BLD Pharm, China). All other chemicals used in the study were purchased from Sigma-Aldrich Chemical (St. Louis, MO, USA) unless specifically stated.

### Animals and ethical approval

In the study, a total of 35 male Sprague-Dawley rats, obtained from the Atatürk University Medical Experimental Application and Research Center, were divided into five groups, with seven animals in each group. The rats were adapted to the environment 7 days prior to the experiment. Animal care and experimental procedures were conducted following the animal research guidelines of the National Institutes of Health and approved by the Local Ethics Committee for Animal Experiments of Atatürk University (Date: 2023\07, Decision Protocol No. 105). Food and water were provided *ad libitum* during the study.

The groups were as follows.

Control group: Rats were given Tween 80 by oral gavage for 14 days.

DINP group: Rats received DINP diluted with Tween 80 at a dose of 200 mg/kg body weight orally by gavage for 14 days (14, 15).

RUT\CD complex group: Rats were given 200 mg/kg body weight of RUT\CD complex by oral gavage in a 1:1 ratio for 14 days (16).

DINP+RUT\CD complex 100 group: Rats were given DINP reconstituted with tween 80 by oral gavage for 14 days, and 30 min later, RUT\CD complex 100 mg/kg body weight in a 1:1 ratio.

DINP+RUT\CD complex 200 group: Rats were given DINP reconstituted with tween 80 by oral gavage for 14 days, and 30 min later, RUT\CD complex 200 mg/kg body weight in a 1:1 ratio.

The rats were sacrificed 24 hr after the end of oral administration under mild sevoflurane anesthesia. Testicular tissue was used for biochemical and histopathological examinations, and epididymal tissue was used for spermatological analysis.

### Preparation of the RUT\CD inclusion complex

The RUT\CD complex was obtained by the method used in the previous study. RUT was complexed with β-CD in a 1:1 molar ratio. 5 g RUT and 5 g β-CD were mixed continuously for approximately 45 min, incubated at 37 °C for one hour, and given fresh to the animals (17).

### ELISA analysis

Testicular tissues were pulverized in liquid nitrogen. The tissues were then reconstituted 1:30 with PBS (pH: 7.4) in Eppendorf tubes and homogenized in a homogenizer (Tissue Lyser II, Qiagen, The Netherlands) using stainless steel balls. Homogenates were centrifuged at 3000 RPM for 20 min and the supernatants were used to determine the levels of MDA, GSH, SOD, CAT, GPx1, NF-κB, TNF-α, IL-1β, IL-6, Cox, Inos, Bax, Bcl-2, and Caspase. The levels of these markers were determined using commercial kits (YL Biont. SOD Catalog No: YLA0115RA, GSH Catalog No: YLA0121RA, MDA Catalog No: YLA0029RA, CAT Catalog No: YLA0123RA, GPx1 Catalog No: YLA0120RA), NF-κB (SunRed, Catalog No: 201-11-5141), TNF-α (YL Biont, Catalog No: YLA0118RA), IL-1β (YL Biont, Catalog No: YLA0030RA), IL-6 (YL Biont, Catalog No: YLA0031RA), COX-2 (YL Biont, Catalog No: YLA0104RA), Inos (YL Biont, Catalog No: YLA0266RA), Bax (YL Biont, Catalog No: YLA0122RA), Bcl-2 (YL Biont, Catalog No: YLA0086RA) and Caspase-3 (YL Biont, Catalog No: YLA1731RA) levels according to the manufacturer’s instructions. The absorbance of the color formed as a result of the analysis was measured at 450 nm wavelength, and calculations were made according to the graphs obtained from the standards of the kits. 

### RT-PCR analysis

From the testicular tissues pulverized as described previously, 100 mg was weighed into sterile microcentrifuge tubes, and 1 ml of QIAzol Lysis Reagent (79306; Qiagen) was added. The tissues were then crushed in a homogenizer for one minute, 200 µl of chloroform was added, and the mixture was incubated for three minutes with vortexing. After incubation, the homogenates were centrifuged at 12000 RPM and 4 °C for 15 min. After centrifugation, the clear part in the uppermost part of the 3 phases was transferred to new tubes, and 500 µl isopropanol was added. The mixture was incubated for 10 min by vortexing and centrifuged at 12000 RPM and 4 °C for 10 min, and isopropanol was removed. The resulting pellet was washed with 1 ml of 75% ethanol. Ethanol was then removed, and RNAs were solubilized with DNase\RNase-free water. The total RNAs obtained were translated into cDNAs using the High-Capacity cDNA Reverse Transcription Kit (Applied Biosystems™ Cat: 4368814, USA). All procedures were performed according to the manufacturer’s instructions. The resulting cDNAs were mixed with iTaq Universal SYBR® Green Supermix (2x), DNase\RNase-free water, and primers with sequences given in Table 1. This mixture was then subjected to reaction in ROTOR-GENE Q (Qiagen, Germany) at 95 °C for 30 sec (1 cycle), 95 °C for 5 sec (40 cycles), 60 °C for 30 sec (40 cycles). After the cycles were completed, genes were normalized to β-actin by the 2-ΔΔCT method. Nrf-2, HO-1, eIF2-α, ATF-4, p53, and Apaf 1 mRNA transcript levels were analyzed by the RT-PCR method.

### Western blot analysis

Protein isolation was performed in the tissue samples obtained from the experimental groups in order to show the amount of protein encoded by the related genes (Bax and Bcl-2). The Qiagen Allpreb protein isolation kit was used for protein isolation. Isolation was performed according to the kit procedure. The concentrations of the isolated proteins were determined by Bradford assay.

First, equal concentrations of proteins were run on resolving and stacking gels. The marker was checked. The gel was shaken with PVDF and 100 % ethanol for 10 min. After running, semi-dry blotting was performed, and proteins were transferred to the membrane (PVDF membrane). The membrane was blocked in TBST with 5% BSA (w/v). The membrane was then washed 3 times for 10 min each with TBST. After washing, the appropriate amount of diluted primary antibody was added to the membrane and left to incubate overnight. After this, the membrane was washed again. After washing, the secondary antibody was added, and incubation was performed (Secondary antibody was selected after RT-PCR (Beta Actin). The bands were visualized with Bio-Rad Clarity Max ECL substrate (Bio-Rad, Hercules, USA) and imaged with Bio-Rad Gel Doc XR+ Imaging System (Bio-Rad, Hercules, USA). Densitometric analysis of the blots was performed using the ImageLab program (Bio-Rad, Hercules, USA). At least three replicate measurements were taken from each sample. Bax and Bcl-2 protein levels were also measured by western blot.

### Histopathologic analysis and evaluation

Testicular specimens obtained from rats sacrificed at the end of the experiment were kept in 10% formalin for 48 hr for fixative purposes. The tissues were dehydrated by passing through an ascending series of ethyl alcohol in accordance with the routine tissue monitoring procedure and then cleaned in xylol. Paraffin blocks were then obtained from testicular tissues subjected to paraffin infiltration. The hematoxylin and eosin (H&E) stained sections were then replaced with sections that were covered with Entellan using coverslips and examined using a binocular Olympus Cx43 light microscope (Olympus Inc., Tokyo, Japan) and photographed with an EP50 camera (Olympus Inc., Tokyo, Japan). Histological changes were evaluated and scored in a blinded manner. According to the Johnson score; 10 points: normal seminiferous tubules and complete spermatogenesis, 9 points: many spermatozoa, irregular tubular germinal epithelium, 8 points: few spermatozoa, 7 points: no spermatozoa, many spermatids, 6 points: no spermatozoa, few spermatids, 5 points: no spermatozoa or spermatids, 4 points: few spermatocytes, 3 points: presence of spermatogonium only, 2 points: no germ cells, presence of Sertoli cells, 1 point: no cells and indicates tubular sclerosis. According to the Cosentino Score; 1 point: Regularly organized germinal cells and normal testicular structure, 2 points: less regular, less organized germinal cells and tightly packed seminiferous tubules, 3 points: less regular, disorganized germ cells and tightly packed seminiferous tubules, 4 points: coagulative necrosis of germ cells and tightly packed seminiferous tubules (18, 19).

### Immunohistopathologic examination

Sections of 3 µm-thick testicular tissues were prepared for immunostaining after passing through xylene and a decreasing alcohol series. To elicit antigens, the sections were kept in citrate buffer at increasingly high temperatures. They were then placed in 3% hydrogen peroxide for 10 min. Sections with inhibited endogenous peroxidase activity were washed with phosphate-buffered saline (PBS) and then treated with protein block for five minutes. The primary antibody (Nitrotyrosine Antibody (sc-32757, PE, Santa Cruz Biotechnology, Dallas, USA) diluted with PBS was dropped onto the sections and kept in the refrigerator at +4 °C overnight. The sections were then treated with secondary antibody and Strepto Biotin separately for 30 min. After each treatment, the sections were washed with PBS 3 times for five minutes each, and DAB solution was dripped onto the sections and allowed to incubate until a brown color appeared. The sections were treated with Harris’s Hematoxylin for five minutes. Tissues were first kept in alcohol series and xylene, and then covered with Entellan. Testicular tissue 3-nitrotyrosine (3-NT) was measured immunohistochemically. It was analyzed under a light microscope at 40x magnification, and 1000 cells from each tissue section for each group were counted according to 3-NT staining positivity.

### Semen analysis

After the rats were sacrificed, the excised testicular tissue was separated from the epididymis. The cauda epididymis was trimmed in 5 ml physiological saline heated to 35 °C and incubated for 5 min. The sperm fluid obtained was used for semen analysis. For the determination of total motility in semen, a slide was placed on a light microscope (Primo Star; Carl Zeiss) with a heating plate. Ten μl of the obtained seminal fluid was dropped onto the slide and covered with a coverslip. Three different microscope fields were examined at 400X magnification, and the final score was scored as a percentage. For sperm density determination, 5 μl of the semen sample was taken into an Eppendorf tube, 995 μl of eosin solution was added, and the mixture was vortexed at 1000 g for 15 sec. 10 μl of the mixture was transferred to a Thoma slide. Sperm counts were calculated using a light microscope (Primostar, Zeiss Co.) set to 400X magnification. For the dead spermatozoa ratio and abnormal sperm ratio, 10 μl of semen and 10 μl of eosin dye (5%) were mixed on the slide with a coverslip, smeared, and dried. For dead spermatozoa, 200 sperm per slide were examined by light microscopy. Sperm cells were classified as dead according to the staining of the head. For abnormal rates of spermatozoa, a total of 200 spermatozoa were evaluated on the same slide, and abnormal rates were calculated as percentages (20).

Sperm DNA damage rate was determined by fluorescence microscopy (Axioscope A1, Zeiss, Germany) using acridine orange stain. 10-μl epididymal sperm samples were frozen and dried on a slide. They were then fixed in Carnoy’s solution (1:3 glacial acetic acid and methanol) for two hours. The slides were then washed with distilled water and stained in freshly prepared acridine orange solution for 5–10 min. The stained slides were washed with distilled water. Randomly selected 200 spermatozoa from each sample were analyzed. Red-colored spermatozoa were considered as DNA-damaged spermatozoa, and the results were calculated as a percentage.

### Statistical analysis

SPSS 26.0 program was used for the evaluation of the data. Kruskal Wallis test was used for the analysis of variance between groups for the semiquantitative data obtained in histopathologic and immunohistochemical examinations and Mann Whitney U test was used for the pairwise comparison of the groups. Biochemical, RT-PCR analyses and western blot parameters were analyzed by one-way analysis of variance, One-Way ANOVA test. Comparison of the groups was performed with Tukey’s test. Results are expressed as mean ± standard deviation (SD).

## Results

### Oxidative stress results

**Figure 1:**
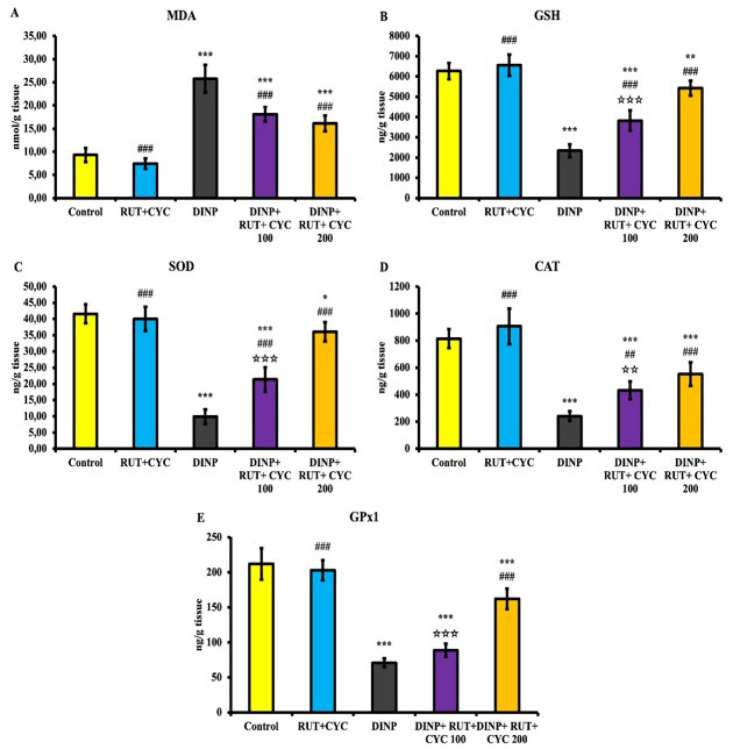
Oxidative stress levels in testicular tissue of rats

Oxidant status of the experimental groups is presented in Figure 1. Accordingly, it is seen that the MDA level increased significantly in the DINP group, whereas MDA level decreased dose-dependently in treatment groups. In addition, GSH level, SOD and CAT levels and GPx1 levels were significantly decreased in DINP groups (*P*<0.001). In the treatment groups, enzymatic and non-enzymatic antioxidant system elements increased dose-dependently.

### Nrf2, HO1 mRNA transcript levels

**Figure 2:**
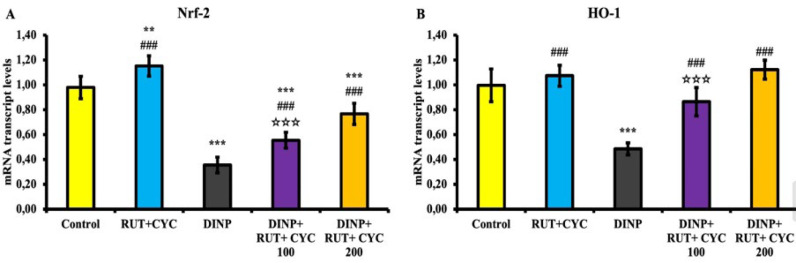
Nrf-2 and HO1 mRNA transcript levels in rat testicular tissue

Nrf-2\HO-1 mRNA transcript levels, one of the common pathways used in the evaluation of oxidative stress, are shown in Figure 2. Nrf-2 levels were significantly increased in the RUT\CD group and decreased in the DINP group (*P*<0.001). It was determined that Nrf-2 level increased dose-dependently in the treatment groups (Figure 2 A). When HO-1 level was analyzed, it was found to be expressed higher in RUT\CD group and DINP+ RUT\CD group compared to other groups. In the DINP group, it was significantly decreased compared to the other groups (*P*<0.001) (Figure 2 B).

### eIF2-α and ATF4 mRNA transcript levels

**Figure 3:**
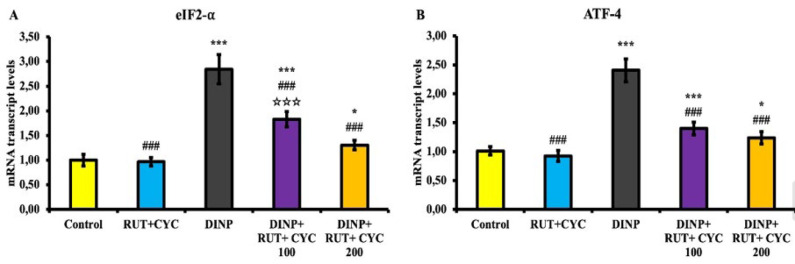
eIF2-α and ATF4 mRNA transcript levels in testicular tissue of rats

The mRNA transcript levels of eIF2-α\ATF4, one of the endoplasmic reticulum stress pathways, are shown in Figure 3. Accordingly, eIF2-α\ATF4 levels were statistically increased in the DINP-treated group compared to the other groups (*P*<0.001). On the other hand, eIF2-α\ATF4 mRNA transcript levels decreased in the treatment groups (*P*<0.001) (Figure 3).

### Inflammation analysis results

**Figure 4:**
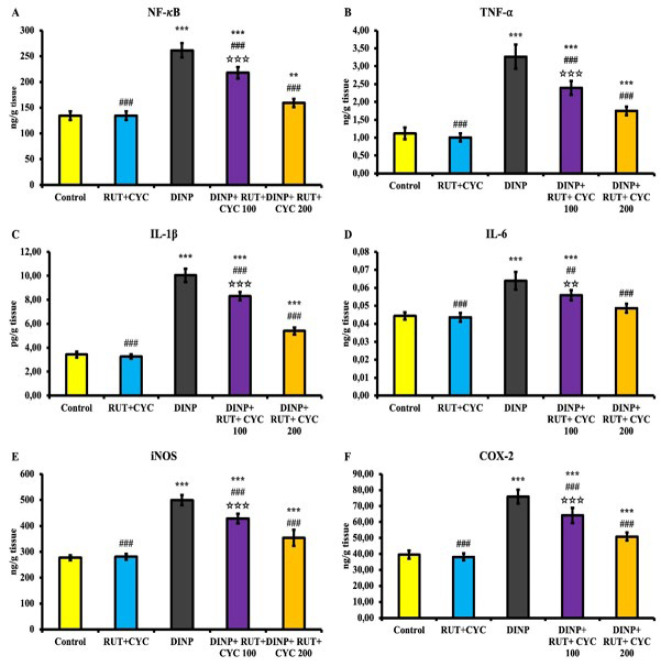
Results of inflammation marker analyses in testicular tissue of rats

Graphs of NF-κB, IL-1β, TNF-α, IL-6, Inos and Cox-2 levels are presented in Figure 4. Accordingly, it was determined that all inflammation parameters were statistically increased in the DINP groups compared to the other groups (*P*<0.001). In the treatment groups, these inflammation parameters were statistically decreased compared to the DINP group (*P*<0.001).

### Apoptosis findings

**Figure 5:**
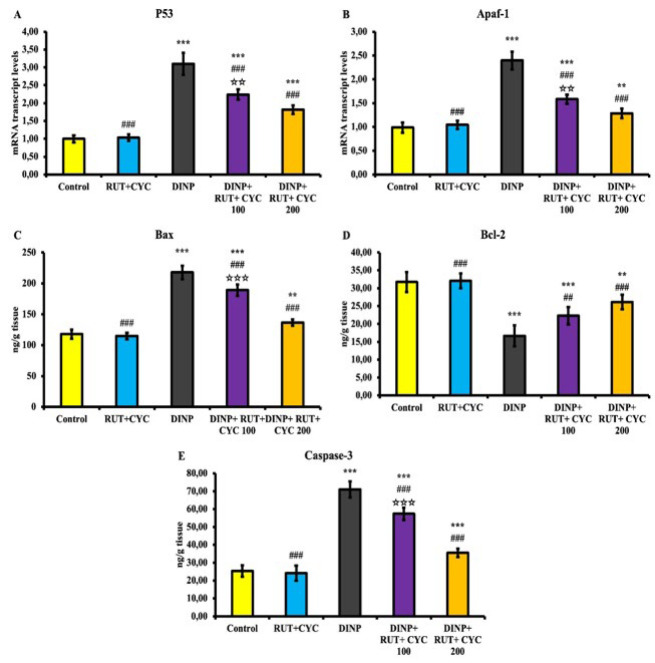
Apoptosis findings in testicular tissue of rats

**Figure 6:**
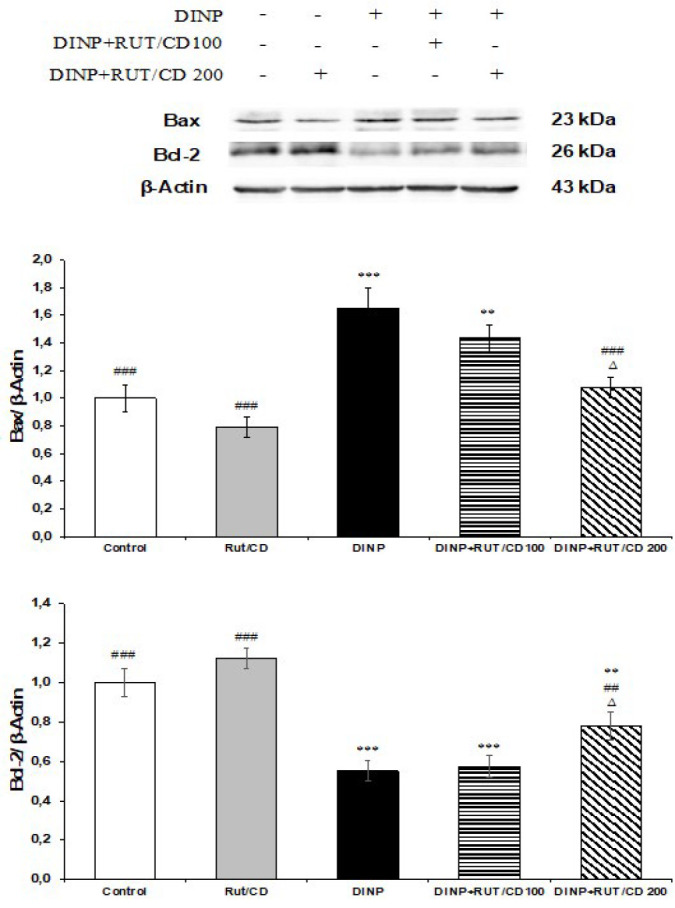
Western blot analysis results in testicular tissue of rats

Apoptosis markers p53, Apaf-1, Bax, Bcl-2 and Caspase-3 levels are presented in Figure 5. DINP administration caused an increase in p53, Apaf-1, Bax and Caspase-3 levels and a decrease in Bcl-2 mRNA transcript levels. In addition, DINP administration caused a decrease in Bcl-2 level. RUT/CD administration following DINP administration inhibited apoptosis in a dose-dependent manner. Western blot analysis of Bax and Bcl-2 levels is shown in Figure 6. The results showed that DINP treatment caused an increase in Bax level and a decrease in Bcl-2 level in testicular tissue (*P*<0.001). In DINP+RUT/CD groups, there was a significant decrease in Bax level and a significant increase in Bcl-2 level compared to the DINP-administered group (*P*<0.001).

### Testicular weights and sperm density, motility, dead and abnormal sperm ratio and sperm DNA damage analysis results

Testicular weight and sperm parameter results are presented in Table 2. There was no statistically significant difference between the groups in terms of testicular weight. Total motility value, epididymal spermatozoon density, percentage of dead spermatozoa and abnormal spermatozoa did not differ between the groups (*P*>0.05). DNA damage was highest in the DINP group and decreased in a dose-dependent manner in the RUT/CD groups following DINP administration (*P*<0.001).

### Results of histopathologic evaluation

**Figure 7:**
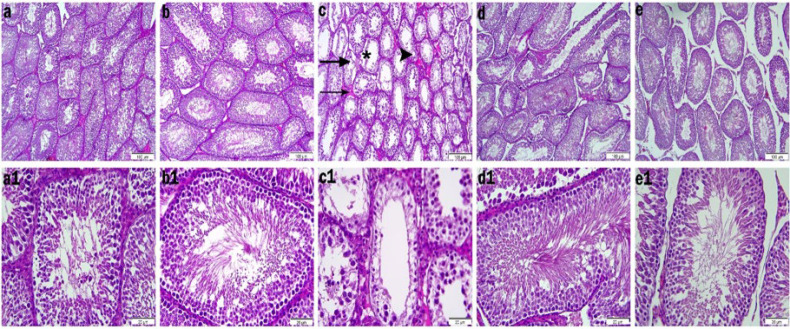
Light microscopic images of testicular tissues of rats

Histopathologic changes in testicular tissue are shown in Figure 7. In the control group, the basement membranes of the seminiferous tubules were intact and spermatogenic cells including spermatogonia, primary spermatocytes, spermatids could be distinguished from germ cells and there were abundant spermatozoa cells from the lumen. In addition, tubule structures were normal and Leydig cells in the interstitial space were smooth (Figure 7a, a1). The slides in the RUT\CD group showed a morphologic structure close to the control group (Figure 7b, b1). In the DINP treated group, degenerated seminiferous tubules with atrophic structure were observed. Most of the germ cells were absent due to a large amount of shedding and interruptions in spermatogenesis. Vacuolization and some necrotic cells were observed in some of the spermatogenic cells in DINP-treated rats. Also, when the scores given in Table 3 were analyzed, there was a decrease in the Johnsen score in the DINP group compared to the other groups, while there was an increase in the cosentine score. In DINP+RUT\CD 100 and DINP+RUT\CD 200 groups, seminiferous tubule structures were improved, and tubule germ epithelium was close to normal. When the scores of DINP+RUT\CD 100 and DINP+RUT\CD 200 groups were analyzed, there was an increase in johnsen score and a decrease in cosentine score compared to DINP group only.

### Immunohistochemical results

**Figure 8:**
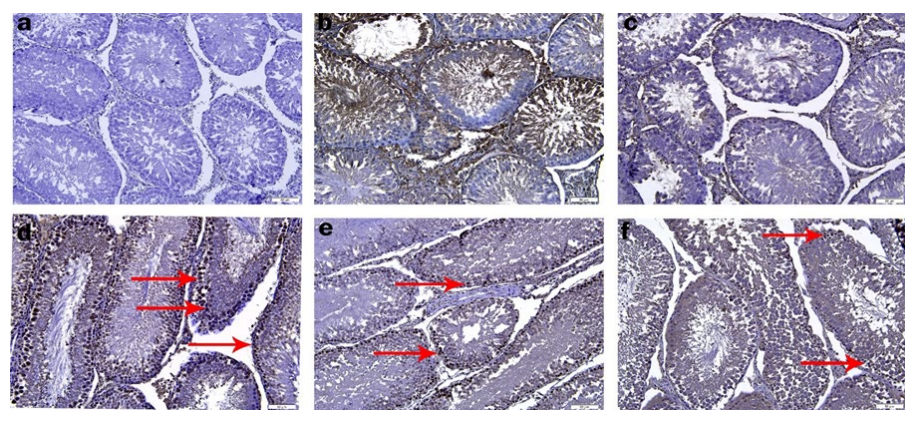
Immunohistochemical staining showing 3-nitrotyrosine (3-NT) expression in testicular tissue of rats

The expression results of 3-NT, one of the primary antibodies we used to determine NO load in the testis using immunostaining, are presented in Figure 8 and Table 3. In the control and RUT\CD group, 3-NT positive expression was weak and there was no significant difference between them (Figure 8b, c). 3-NT positive expression was significantly increased in DINP group compared to the other groups and control (*P*<0.05) (Figure 8d). In DINP+RUT\CD100 and DINP+RUT\CD200 groups, the number of dark stained cells decreased compared to DINP group and DINP+RUT\CD200 group was more effective (*P*<0.05) (Figure 8e, f).

## Discussion

DINP is a high molecular weight, general purpose plasticizer used primarily in the manufacture of polymers and consumer products (7). Studies have reported that DINP has negative effects on the reproductive system (21). RUT is one of the most potent antioxidant compounds, but has low absorption and bioavailability in the gastrointestinal tract due to its limited water solubility (13, 22). Cyclodextrins increase the water solubility of active substances. In this study, oxidative stress, apoptosis, inflammation and sperm DNA damage were induced in DINP-administered rats and protective effects of RUT\CD complex were determined.

Excessive cellular ROS production disrupts redox balance and decreases antioxidant enzyme activities, leading to cytotoxicity and genotoxicity (23). Increased ROS results in oxidative stress due to inadequate and/or suppression of antioxidant defense mechanisms (24).

Oxidative stress is indicated as the basic mechanism of tissue damage caused by toxic substances (25). Enzymes such as SOD, CAT and GPx, which provide defense against ROS in the body, are the first line of defense against oxidative stress. The second line of defense is GSH levels (26). Toxic substances are one of the main causes of testicular oxidative stress (27). DINP exposure causes an increase in oxidative stress levels in kidney tissue of mice (28). Previous studies have reported that phthalates cause oxidative stress in the male reproductive system (29). In other studies, oral DINP administration caused an increase in oxidative stress levels in the liver and kidneys (30). In the present study, it was observed that DINP administration caused an increase in MDA level in testicular tissue of rats. However, it was determined that RUT\CD administration inhibited the increased MDA level. In addition, DINP administration caused a decrease in SOD, CAT, GPx1 activity and GSH level, while it was determined to increase with RUT\CD administration.

Nrf-2 is a critical transcription factor induced by oxidative stress and responsible for the activation of several antioxidant genes (31). Nrf-2, which is translocated to the nucleus, regulates the activation of endogenous antioxidant genes such as HO-1 (32). The enzymatic activity of HO-1, one of the Nrf-2 target genes, may be highly effective in many tissues that protect against cellular oxidative stress caused by high ROS (33). It has been reported that phthalates increase the levels of Nrf-2 and HO-1 as well as testicular, kidney, and cardiac tissue (34-37). Rats in the DINP group had lower levels of Nrf-2 and HO-1 mRNA transcripts in the testicular tissue than rats in the other experimental groups. Nrf-2 and HO-1 levels were highest in the RUT\CD alone group. According to this determination, RUT/CD administration reduced oxidative stress and increased antioxidant activity, hence preventing ROS. Compared to the DINP-alone-treated group, Nrf-2 and HO-1 mRNA transcript levels rose in the DINP+RUT\CD-treated groups. This was regarded as RUT\CD administration inhibiting DINP-induced oxidative stress.

The endoplasmic reticulum (ER) is a vital organelle that performs a variety of functions essential for proper cellular activity and survival (38). Accumulation of unfolded and/or misfolded proteins in the ER triggers the ER stress response, the unfolded protein response (39). ER stress is associated with parameters such as glucose-regulated protein 78 (GRP-78), activating transcription factor 4 (ATF-4), eukaryotic initiation factor 2 alpha (EIF2-A), caspase-3, caspase-9 and CCAAT/enhancer binding protein (CEBP) (40). The ER stress pathway is one of the underlying mechanisms eliciting DEHP toxicity (41). In a study, it was reported that DEHP can induce testicular apoptosis in mice through ER stress (42). Another study reported that DEHP induced apoptosis in testicular cells through the PERK-eIF2α-ATF4-CHOP pathway mediated by mitochondrial dysfunction (43). According to the present study, the administration of DINP may activate the eIF2-α/ATF-4 pathway by raising oxidative stress, which causes ER stress and apoptosis. However, by reducing oxidative stress, RUT-CD administration inhibited this pathway, which was observed as a decrease in apoptosis.

Apoptosis is the mechanism that causes DNA fragmentation and is characterized by chromatin breakage during planned cell death (44). Apoptosis is a programmed cell death that promotes homeostasis in the body through the balance between pro-apoptotic such as Bax, Caspase-3 and anti-apoptotic such as Bcl-2 (45). ROS is considered to be one of the main mechanisms in triggering apoptosis. Previous studies indicate that oxidative stress increases in the testes due to various toxic agents and apoptosis is induced accordingly (46). Phthalates are reported to induce apoptosis in germ cells and testes (47). It has also been observed that DINP causes ovarian germ cells to undergo apoptosis (48). It has been reported to induce DINP-induced ROS and cause apoptosis in oocyte cells in pigs (49). In the present study, Bax and Caspase-3 levels were significantly increased in DINP-administered groups, whereas Bcl-2 levels were significantly decreased compared to the other groups. In RUT\CD groups, Bax and Caspase-3 levels decreased, and Bcl-2 levels increased in a dose-dependent manner. This is thought to be induced by ROS.

NF-κB, an important marker of inflammatory damage, plays a key role in many biological processes such as regulation of proinflammatory mediators, expression of chemokines, activation of cytokines, immune response and cell differentiation (45). Once activated, NF-κB causes the expression of cytokines that are crucial for inflammation, including TNF-α, IL-6, and IL-1β (50). Expression of Inos and Cox-2 proteins is regulated by NF-κB (51). In addition, spermatogenesis and the function of Sertoli cells in the testes are controlled by NF-κB (52). DBP is reported to induce apoptosis of Sertoli cells and NF-κB activation (53). Phthalates are also reported to induce inflammation in various tissues (54, 55). DINP is reported to activate NF-κB in Balb/c mice through the mediation of the NF-κB signaling pathway (56). In the present study, it was observed that DINP administration caused an increase in NF-κB activation in the testicular tissue of rats. The increase in the level of other inflammation mediators as a result of activated NF-κB was interpreted as DINP triggered inflammation in testicular tissue in rats. It has been reported that RUT\CD complex causes a decrease in inflammatory mediators in lambda-cyhalothrin-induced hepatotoxicity and nephrotoxicity (16). In this study, it was observed that RUT\CD treatment decreased DINP-induced inflammation. This is thought to be due to the antioxidant properties of RUT as well as the increased absorption and bioavailability of CD in water.

The testes are responsible for producing viable sperm and steroid hormones for sexual and reproductive function. Reduced anogenital distance, retained nipples, hypospadias, undescended testes, epididymal agenesis, and low sperm count are all consequences of phthalates’ negative effects on the male reproductive system in male rats (57). In newborn male rats, phthalate exposure has been shown to reduce testicular testosterone levels, leading to down-regulation of some steroidogenesis-related genes (58). In a study, it was reported that DINP administration altered testicular histoarchitecture in rats (57). Degenerated seminiferous tubules with an atrophic structure, large-scale shedding of germ cells and interruptions in spermatogenesis were observed in the DINP group. When Johnsen score was evaluated, it was observed that DINP application decreased while Cosentine score increased. When the expression level of 3-nitrotyrosine (3-NT) was analyzed, it was observed that DINP administration caused an increase in the expression level.

Free radicals induce sperm oxidative stress, which is associated with poor sperm function (59). Various toxicant have been reported to cause infertility by reducing sperm quality (60). In the present study, sperm motility value, epididymal sperm density, testicular weights, plasma membrane integrity and abnormal sperm rate did not show a statistically significant difference between the groups. However, the sperm DNA damage rate was higher in DINP-treated groups compared to the other groups, while this rate decreased dose-dependently in DINP+RUT\CD-treated groups. This was thought to occur after DINP-induced oxidative stress increase triggered ER stress and apoptosis.

**Table 1:** Primer sequences of rats

Gen	Sequence (5’-3’)	Length (bp)	Accession No
Nrf2	F: TTTGTAGATGACCATGAGTCGC R: TCCTGCCAAACTTGCTCCAT	161	NM_031789.2
HO-1	F: ATGTCCCAGGATTTGTCCGA R: ATGGTACAAGGAGGCCATCA	144	NM_012580.2
P53	F: GCGCTTCGAGATGTTCCGA R: AGACTGGCCCTTCTTGGTCT	121	NM_030989.3
Apaf-1	F: ACCTGAGGTGTCAGGACC R: CCGTCGAGCATGAGCCAA	192	NM_023979.2
eIF2-	F: AGACCTGGATATGGTGCCTA R: CCTTCGTAACCATAGCAAGC	182	NM_019356.1
ATF-4	F: CTTCCTGAACAGCGAAGTGT R: ATAGCCAGCCATTCTGAGGA	171	NM_024403.2
-Actin	F: CAGCCTTCCTTCCTGGGTATG R: AGCTCAGTAACAGTCCGCCT	360	NM_031144.3

All data are expressed as mean ± SD. Statistical significance (Control vs others: *P<0.05, **P<0.01, ***P<0.001, DINP vs others: #*P*<0.05, ###*P*<0.01, ####*P*<0.001, DINP+RUT\CD100 vs DINP+RUT\CD200: *P<0.05, **P<0.01, ***P<0.001).

All data are expressed as mean ± SD. Statistical significance (Control vs others: *P<0.05, **P<0.01, ***P<0.001, DINP vs others: #*P*<0.05, ###*P*<0.01, ####*P*<0.001, DINP+RUT\CD100 vs DINP+RUT\CD200: *P<0.05, ***P<0.01, ****P<0.001).

All data are expressed as mean ± SD. Statistical significance (Control vs others: *P<0.05, **P<0.01, ***P<0.001, DINP vs others: #*P*<0.05, ###*P*<0.01, ####*P*<0.001, DINP+RUT\CD100 vs DINP+RUT\CD200: *P<0.05, ***P<0.01, ****P<0.001).

All data are expressed as mean ± SD. Statistical significance (Control vs others: *P<0.05, **P<0.01, ***P<0.001, DINP vs others: #*P*<0.05, ###*P*<0.01, ####*P*<0.001, DINP+RUT\CD100 vs DINP+RUT\CD200: *P<0.05, ***P<0.01, ****P<0.001).

All data are expressed as mean ± SD. Statistical significance (Control vs others: *P<0.05, **P<0.01, ***P<0.001, DINP vs others: #*P*<0.05, ###*P*<0.01, ####*P*<0.001, DINP+RUT\CD100 vs DINP+RUT\CD200: *P<0.05, ***P<0.01,****P<0.001).

All data are expressed as mean ± SD. Statistical significance (Control vs others: *P<0.05, **P<0.01, ***P<0.001, DINP vs others: #*P*<0.05, ###*P*<0.01, ####*P*<0.001, DINP+RUT\CD100 vs DINP+RUT\CD200: ∆*P*<0.05, ∆∆∆*P*<0.01, ∆∆∆∆*P*<0.001).

**Table 2:** Reproductive parameter results of rats in the experimental group

	Control	DINP	RUT\CD	DINP+RUT\CD200	DINP+RUT\CD100
Total testis weight (mg)	3.09±0.11	3.05±0.10	2.94±0.14	3.03±0.12	2.87±0.07
Total motility (%)	82.85±2.14	78.57±1.42	80.71±2.02	79.28±2.51	79.16±1.53
Density (x10^6^)	22.71±1.64	18.28±1.37	20.28±1.68	20.66±2.47	18.71±0.80
Dead spermatozoa rate (%)	21.57±1.83	31.14±1.98	29.33±1.26	27.14±2.41	28.00±2.25
Anormal spermatozoa rate (%)	7.42±0.64	8.42±0.61	7.28±0.56	8.33±0.80	8.28±0.52
DNA damage (%)	12.83±0.70^a^	17.33±2.16^c^	12.01±1.41^a^	13.83±1.47^ab^	14.16±2.37^b^

Superscript letters (a, b, c) indicate the difference between groups. *P*<0.001.

Normal testicular sections from the control (a, a1) and RUT\CD (b, b1) groups, shrunken and atrophic seminiferous tubules (thick arrow), germinal epithelial degeneration and desquamation of spermatogenic cells (asterisk) in the DINP (c, c1) treated group, vacuolization in the seminiferous tubule (arrow head), oedema formation in the interstitial space (thin arrow), near normal sections from DINP+RUT\CD100 (d, d1) DINP+RUT\CD200 (e, e1) groups (HE, ×100, ×400).

**Table 3:** Histopathological and immunohistochemical evaluations of rats

Parameters	Control	RUT\CD	DINP	DINP+RUT\CD200	DINP+RUT\CD100
Johnsen score Cosentine score 3-nitrotirozini(3-NT)	9,28±0,75^#^ 1,28±0,48^#^ 109,4±16,09^#^	9,71±0,48 1,14±0,37 95±10,13	4,28±0,95* 3,28±0,48* 376,1±22,9*	8±0,81*^#^ 1,57±0,53^#^ 137,14±8,05*^#+^	7,57±0,97*^#^ 2±0,57^#^ 168,7±13,9*^#^

Data are mean ± SD. * (*P*<0.05) compared to control group, # (*P*<0.05) compared to DINP group, + (*P*<0.05) compared to DINP+ RUT\CD100 group

(a) Negative control, mild staining (×200) in Control (b) and RUT\CD (c) group. (d) Diffuse 3-NT staining (×200) (arrows) in DINP group. (e) DINP+ RUT\CD100 moderate 3-NT staining (×200) (arrows). (f) DINP+ RUT\CD200 low 3-NT staining (×200) (arrows) close to control group.

## Conclusion

The present study investigated the effects of RUT\CD complex, a potent antioxidant, against DINP, a common environmental pollutant. When the findings were evaluated, it was determined that DINP application triggered oxidative stress, inflammation and apoptosis in rats, while RUT\CD complex inhibited this increase in oxidative stress, inflammation and apoptosis. In addition, DINP treatment caused histological changes in the testes, whereas RUT\CD treatment preserved the testicular histology. There was no statistically significant difference between the groups in spermatologic analysis. It was interpreted that DINP application in rats was not affected by the inability to pass the blood testicular barrier in a short time and the prolonged duration of spermatogenesis. In future studies, it is thought that longer-term applications should be performed in order to see the effect of DINP more clearly.

## Data Availability

The corresponding author may provide the study’s data upon reasonable request.

## Declaration

We have not used any AI tools or technologies to prepare this manuscript.
